# Investigations of microbiota composition and neuroactive pathways in association with symptoms of stress and depression in a cohort of healthy women

**DOI:** 10.3389/fcimb.2024.1324794

**Published:** 2024-07-02

**Authors:** Zahra Bashir, Luisa W. Hugerth, Maria Christine Krog, Stefanie Prast-Nielsen, Gabriella Edfeldt, Fredrik Boulund, Simon Rønnow Schacht, Inge Tetens, Lars Engstrand, Ina Schuppe-Koistinen, Emma Fransson, Henriette Svarre Nielsen

**Affiliations:** ^1^ Department of Obstetrics and Gynecology, Slagelse Hospital, Slagelse, Denmark; ^2^ The Recurrent Pregnancy Loss Unit, Dept. of Fertility, The Capital Region, Copenhagen University Hospitals, Rigshospitalet, Copenhagen, Denmark; ^3^ Dept. of Obstetrics and Gynecology, Hvidovre Hospital, Hvidovre, Denmark; ^4^ Department of Microbiology, Tumor and Cell Biology, Karolinska Institutet, Stockholm, Sweden; ^5^ Department of Medical Biochemistry and Microbiology, Science for Life Laboratory, Uppsala University, Uppsala, Sweden; ^6^ Department of Clinical Immunology, Copenhagen University Hospital, Rigshospitalet, Copenhagen, Denmark; ^7^ Science for Life Laboratory, Karolinska Institutet, Stockholm, Sweden; ^8^ Department of Nutrition, Exercise and Sports, University of Copenhagen, Copenhagen, Denmark; ^9^ Department of Women’s and Children’s Health, Uppsala University, Uppsala, Sweden; ^10^ Department of Clinical Medicine, University of Copenhagen, Copenhagen, Denmark; ^11^ Department of Obstetrics and Gynecology, Hvidovre Hospital, Copenhagen, Denmark

**Keywords:** depressive symptoms, diet, microbiome, tryptophan, perceived stress, shotgun sequencing, proteobacteria, (microbiota-)gut-brain axis

## Abstract

**Background:**

Despite mounting evidence of gut-brain involvement in psychiatric conditions, functional data remain limited, and analyses of other microbial niches, such as the vaginal microbiota, are lacking in relation to mental health. This aim of this study was to investigate if the connections between the gut microbiome and mental health observed in populations with a clinical diagnosis of mental illness extend to healthy women experiencing stress and depressive symptoms. Additionally, this study examined the functional pathways of the gut microbiota according to the levels of psychological symptoms. Furthermore, the study aimed to explore potential correlations between the vaginal microbiome and mental health parameters in young women without psychiatric diagnoses.

**Methods:**

In this cross-sectional study, 160 healthy Danish women (aged 18-40 years) filled out questionnaires with validated scales measuring symptoms of stress and depression and frequency of dietary intake. Fecal and vaginal microbiota samples were collected at the beginning of the menstrual cycle and vaginal samples were also collected at cycle day 8-12 and 18-22. Shotgun metagenomic profiling of the gut and vaginal microbiome was performed. The Kyoto Encyclopedia of Genes and Genomes (KEGG) was used for functional profiling and 56 Gut Brain Modules were analyzed in the fecal samples.

**Results:**

The relative abundance in the gut of the genera *Escherichia*, *Parabacteroides*, and *Shigella* was higher in women with elevated depressive symptoms. Women with high perceived stress showed a tendency of increased abundance of *Escherichia*, *Shigella*, and *Blautia*. Amongst others, the potentially pathogenic genera, Escherichia and Shigella correlate with alterations in the neuroactive pathways such as the glutamatergic, GABAeric, dopaminergic, and Kynurenine pathways. Vaginosis symptoms were more prevalent in women reporting high levels of stress and depressive symptoms.

**Conclusions:**

The findings of this study support the concept of a microbiota-associated effect on the neuroactive pathways even in healthy young women. This suggest, that targeting the gut microbiome could be a promising approach for future psychiatric interventions.

## 1 Introduction

Mental health disorders contribute significantly to the global burden of disease, with major depression affecting more than 300 million individuals worldwide (World Health Organization, 2017; Rehm and Shield, 2019). The link between the gut and the brain has been studied immensely revealing specific microbiota compositions related to several mental health disorders (Skonieczna-żydecka et al., 2018b). Multiple routes of communication exist in the crosstalk between the gut and brain which primarily occurs via the hypothalamic-pituitary-adrenal axis, the autonomic nervous system (ANS), and through metabolites and neurotransmitters also emanating from the gut (Rieder et al., 2017; Malan-Muller et al., 2018; Liu et al., 2023).

About 90% of the adult gut microbiome consists of the phyla Bacteroidetes, Firmicutes, Actinobacteria, Proteobacteria, and Verrucomicrobia, with the most dominant phyla being Firmicutes and Bacteroidetes (Kelly et al., 2016; Winter et al., 2018; Sasso et al., 2023). A decrease in Bacteroidetes can initiate dysbiosis (microbiome imbalance) and inflammation and has been suggested as an indicator of major depression (Levy et al., 2016; Rong et al., 2019) with different alterations of the gut microbiota being reported in patients diagnosed with major depression (Naseribafrouei et al., 2014; Jiang et al., 2015; Cheung et al., 2019; Liu et al., 2020) as well as patients diagnosed with chronic stress, anxiety, irritable bowel syndrome (IBS) and inflammatory bowel disease (IBD) (Molina-Torres et al., 2019; Humbel et al., 2020). Some studies have found a decrease in the Firmicutes/Bacteroidetes (F/B) ratio in depressed individuals (Jiang et al., 2015; Huang et al., 2018; Liu et al., 2020, 2023) while others reported a higher F/B ratio (Naseribafrouei et al., 2014; Zheng et al., 2016; Lai et al., 2019; Rong et al., 2019). Actinobacteria has also been shown to be more abundant in the gut microbiota of depressed patients compared with controls (Zheng et al., 2016; Chen et al., 2018; Chung et al., 2019; Lai et al., 2019; Rong et al., 2019), potentially more often in women than men (J. J. Chen et al., 2018). The genera *Bifidobacterium* (phylum Actinobacteria), together with *Faecalibacterium* and *Ruminococcus* (Firmicutes) are important for carbohydrate metabolism and have been found to be reduced in depressed individuals (Cheung et al., 2019). In addition, short-chain fatty acids (SCFAs), metabolites produced by bacterial fermentation of fibers (Silva et al., 2020) have been suggested to play a role in the microbiota-gut-brain axis in depressed individuals (Zalar et al., 2018; Silva et al., 2020; Liu et al., 2023). In summary, the findings from previous studies point to a role for the gut microbiome in mental health, however the role of specific genera and their mechanism of action is unclear (Borkent et al., 2022). Whether the alteration in microbiome composition observed in depressed individuals is a cause or consequence of the disease or treatment remains to be determined. To our knowledge, there is a knowledge gap as no data exists on the microbiome composition in young women with symptoms of stress and depression without a clinical diagnosis of major depression. These data could be valuable in understanding the contribution of the microbiome on the development of mental health problems.

Major depression and IBS are more prevalent in women than men (Warnock and Clayton, 2003). Both estrogen and progesterone possess brain receptors and have the capacity to affect mood and behavior (Saito and Cui, 2018; Guennoun, 2020). While the association between gut microbiota and mental health is well established (Cheung et al., 2019), limited focus has been on the possible link between the vaginal microbiota and mental health. In reproductive-aged women, the vaginal microbiota is often dominated by *Lactobacillus* spp (Gajer et al., 2012; Hugerth et al., 2020; Krog et al., 2022a). Absence of *Lactobacillus* dominance is associated with increased susceptibility to gynecological infections, including the condition bacterial vaginosis (BV) (Green et al., 2015; Norenhag et al., 2019). The occurrence of BV has been related to stress exposure (Culhane et al., 2006; Amabebe and Anumba, 2018) while the potential association between vaginal *Lactobacillus* spp. dominance or dysbiosis and women’s mental health remain to be investigated. However, the vaginal microbiome is highly dynamic and changes during the menstrual cycle, also shown in this cohort (Krog et al., 2022a), and the timing of sampling must be taken into account in all analyses.

Previous studies of the microbiome and mental health have mostly been performed in patients diagnosed with major depression during treatment with antidepressants (Cheung et al., 2019). Knowing that antidepressants *per se* have been shown to alter the gut microbiota (Ait Chait et al., 2020), it remains unclear how previous findings will replicate in a general, healthy population of women. To increase the knowledge on the mental health impact of the microbiome in a non-psychiatric population of women, this study aimed to investigate symptoms of stress and depression in relation to the gut and vaginal microbiome by metagenomic profiling in a cohort of healthy women. Additionally, another aim was to investigate potential functional and metabolic pathways of the gut microbiome.

## 2 Materials and methods

### 2.1 Participants and procedures

This study is part of the MiMens cohort study, designed to investigate the microbiome during the menstrual cycle and in relation to hormonal contraception (Krog et al., 2022a, 2022b). The study enrolled 160 healthy women aged 18 to 40 years at Copenhagen University Hospital, Rigshospitalet, Denmark. Exclusion criteria were current or planned pregnancy and receiving antibiotics or antiviral medication within the previous two weeks. At baseline, study participants completed a comprehensive questionnaire including validated tools described below, and questions about health, medication and lifestyle factors. The baseline questionnaire included questions about gynecological problems during the past 24 hours, including symptoms of BV (“unpleasant vaginal odor” and “vaginal discharge”). Participants also kept records of their bleeding patterns and frequency of intercourse for six weeks after the first sampling. In this study, data were used from the first 28 days, corresponding to an average length of a menstrual cycle.

Informed consent was obtained from all participants. The study was approved by The Regional Ethics Committee (H-17017580) and the Data Protection Agency (RH-2017-280, I-Suite 05825) in the Capital Region of Denmark. All procedures contributing to this work comply with the ethical standards of the relevant national and institutional committees on human experimentation and with the Helsinki Declaration of 1975, as revised in 2008.

#### 2.1.1 Measurement of stress and depression

Stress was measured using the ten-item Perceived Stress Scale (PSS-10), which is a validated questionnaire used for comparisons between groups, not a diagnostic tool (Cohen and Williamson, 1988). The PSS-10 measures the subjective perception of stress during the past month on a 5-point Likert scale (0-4), with a total score ranging from 0 (no stress) to 40 (extreme stress) (Cohen and Williamson, 1988). Based on a national representative sample of the Danish population, a cut-off score of PSS ≥17 was used as an indicator of high-stress (Nielsen et al., 2008).

The Major Depression Inventory (MDI) is a validated questionnaire with ten questions covering the core and additional symptoms of depression in the International Classification of Diseases, Tenth Revision (ICD-10) and Diagnostic and Statistical Manual of Mental Disorders, 4th ed. (DSM-IV) symptoms of depressive illness. It can be used in a clinical setting to assess symptoms of depression and was developed to be used as a diagnostic tool (Olsen et al., 2004). Each question is scored on a 0 to 5 Likert scale, and the score measures the presence of the symptoms during the past two weeks (Olsen et al., 2004; Bech et al., 2015). A total score between 20-50 can be used to define the depressive range (Bech et al., 2015). Due to the few cases fulfilling depression according to the diagnostic tool in this cohort, the 90^th^ percentile was used; MDI total score >18 as the cut-off for elevated symptoms.

#### 2.1.2 Assessment of colon transit time

The Bristol Stool Form Scale, BSFS (Lewis and Heaton, 1997), was filled out at baseline where the participants reported on the stool types, they had during the past two weeks. The reports were categorized into four groups: Normal colonic transit (BSFS 3-4 only); Slow colonic transit (including BSFS 1-2); Fast colonic transit (including BSFS 5-7) and Various transit: both fast and slow (BSFS 1-2 and BSFS 5-7).

#### 2.1.3 Habitual dietary intake

The baseline questionnaire included questions on habitual dietary intake measured in a food frequency questionnaire based on a four-week period, with frequencies given on a 9-point scale from “0 times in the past four weeks” to “>3 times/day for the past four weeks” (Bostanci et al., 2021). The frequency of fiber intake was estimated using six questions on how often fiber-rich products from the following food groups were consumed: rye bread; whole wheat bread; fruit; vegetable dishes; salad; and prepared vegetables. Reporting at least one daily intake of a fiber-rich food was categorized as “daily”, while other responses were categorized as “less often”.

The frequency of sugar intake was estimated using the same 9-point scale on the frequency of consumption of: chocolate milk, juice, soda with sugar, ice cream, biscuits or cookies, sweet bread and rolls, dry cake, cake with filling, and candy. Sugar consumption in the highest quartile (>=22.7 occasions/week) was classified as “daily”; in the middle quartiles as “weekly”; and in the lowest quartile (<=4 occasions/week) was categorized as “seldom/never” (Bostanci et al., 2021).

#### 2.1.4 Sample collection for analyses of gut and vaginal microbiota

Participants were invited to the hospital for gynecological examination and blood sampling at three-time points during their menstrual cycle; during the menses cycle day (CD) 1-3, during the follicular phase CD 8-12, and during the luteal phase CD 18-22 (for further details see (Krog et al., 2022b)). In this study, we analyzed 160 fecal and 160 vaginal samples from the first visit at CD 1-3 from 160 participants, when perceived stress and depressive symptoms were assessed, as previously described (Krog et al., 2022a). For the vaginal samples, all three time-points were taken into account.

The participants received self-collection kits for home sampling of the vaginal and fecal samples and were instructed in sampling by the first-author.

The vaginal samples were collected with FLOQSwabs (Copan Flock Technologies, Brescia, Italy) and put directly into FluidX tubes (Brooks Life Sciences, Chelmsford, MA, USA) containing 0.8 ml DNA/RNA-shield (Zymo Research, Irvine, CA, USA). The participants were instructed to separate the labia majora to minimize external contamination and insert the swab three centimeters inside the vagina and rotate the swab 20 seconds. Fecal samples were collected by the participants at home within 48 hours following their hospital visit using the Zymo’s DNA/RNA-shield Fecal Collection Tube and stored at ambient temperature until the next hospital visit.

#### 2.1.5 DNA extraction, shallow shotgun metagenomic sequencing, and annotation

Samples were extracted with MOBio PowerFecal (Qiagen, Hilden, Germany) automated on QiaCube (Qiagen), with bead-beating in 0.1 mm glass bead plates. Three spaced negative controls and one positive control were included in each extraction. All negative extraction controls had undetectable amounts of DNA, and all positive controls were approved. The DNA concentration of samples and controls was quantified using Quant-iT Picogreen dsDNA Assay (Invitrogen, ThermoFisher Scientific, Carlsbad, CA, USA). Samples were shipped for metagenomic sequencing using BoosterShot technology to CoreBiome (OraSure, Bethlehem, PA, USA) on single-ended 150 bp reads on an Illumina NextSeq 550 with the high output v2 kit. BoosterShot is designed to obtain accurate species-level information from a few reads as described in (Hillmann et al., 2018). On average, 2,398,197 reads were generated per sample (median 2,398,317) of which 41.1% mapped to their curated reference microbial database.

#### 2.1.6 Assessment of hormone levels in blood

Blood was drawn and collected in EDTA-tubes at the three hospital visits (Krog et al., 2022a). The samples were centrifuged and plasma was aliquoted and frozen at -80° degrees Celsius. Estradiol and progesterone levels were measured using the standard automated system (Cobas^®^ 8000 by Roche Diagnostics). The average hormone levels for the three-time points were calculated for each participant.

### 2.2 Statistical analyses and bioinformatics

Descriptive statistics were presented as medians and ranges and categorical data as numbers and percentages. Differences between stress and depressive symptoms groups were assessed using the Mann-Whitney U-test and chi-square test or Fisher’s exact test where appropriate. Univariable logistic regressions were used for the colonic transit time variable and the psychological symptom groups. Factors found to differ between symptomatic and asymptomatic women were adjusted for in downstream analyses.

Fecal microbiota was analyzed based on CoreBiome’s filtered taxonomy file. Because BoosterShot is not optimized for vaginal samples, these were re-annotated using Kraken2 (Wood et al., 2019) on the OptiVag database v.1.0 (Hugerth et al., 2020). Alpha diversity (within sample diversity) was calculated as the observed number of species (richness) as well as Simpson’s inverted index, using the Vegan package (v.2.5-3) in R (v.3.5.2). Functional annotation of fecal metagenomes was performed by CoreBiome.

The vaginal microbiome was classified based on vaginal community dynamics (VCD) during the menstrual cycle (Hugerth et al., 2023). In detail, all samples were first assigned to community state types using Valencia (France et al., 2020). Then, considering CST-I, CST-II and CST-V as eubiotic, participants were classified as “Constant eubiotic” if all their samples were eubiotic, “Menses dysbiotic” if only the first sample was dysbiotic, “Constant dysbiotic” if all samples were dysbiotic and the remaining were classified as “Unstable”.

#### 2.2.1 Microbiota composition and psychological health

To investigate potential associations between composite microbiota variables (fecal diversity and richness, fecal F/B ratio, vaginal diversity, and percent of *Lactobacillus* spp. in the vaginal sample) and psychological health (low/high depressive symptoms, and low/high perceived stress), univariable logistic regressions were performed. *L. iners* was excluded from this analysis since it has been shown to play an ambiguous role in the vaginal tract (Zheng et al., 2021). Multivariable logistic regressions were then applied and adjusted for potential confounders (Sugar intake and bowel/colonic transit time when analyzing gut microbiota; and symptoms of BV (“unpleasant vaginal odor” and “vaginal discharge”) when analyzing vaginal microbiota). Additionally, PSS and MDI scores were analyzed in relation to VCD using ANOVA. The correlation between days with sexual intercourse and PSS and MDI were calculated as Pearson’s product-moment correlation.

#### 2.2.2 Abundance of specific taxa in association with symptoms of stress/depression

Samples were transformed by centered log-ratio before the tests were performed. Before the transformation, a pseudo count corresponding to 0.00001 of a read was added to all values. Differences in the relative abundance of specific genera previously reported in a systematic review to associate with mood (Cheung et al., 2019), were investigated with Mann-Whitney U tests between low/high depressive symptoms and low/high perceived stress, respectively (*Anaerostipes*, *Blautia*, *Alistipes*, *Escherichia*, *Shigella*, *Clostridium*, *Lachnospiraceae*, *Parabacteroides*, *Parasutterella*, *Phascolarctobacterium*, *Streptococcus*, *Bifidobacterium*, *Dialister, Faecalibacterium*, *Ruminococcus*, *Bacteroides*, *Oscillibacter*, *Prevotella*, *Roseburia*, *Actinobacteria*, *Klebsiella* and *Megamonas*).

The median and interquartile range of microbiota composition and abundance of specific taxa per group (low/high depressive symptoms and low/high perceived stress) were illustrated using violin plots (GraphPad Prism version 8.3.0 for Windows, GraphPad Software, La Jolla California USA).

#### 2.2.3 Bacterial functional pathways related to the Microbiota Gut-Brain axis

The functional profile of the bacteria can be investigated by mapping the bacterial DNA reads against the Kyoto Encyclopedia of Genes and Genomes (KEGG). A total of 56 gut-brain modules (GBMs) were identified, each corresponding to a known single neuroactive compound production or degradation process (Valles-Colomer et al., 2019), applied with the package omixer-rpm in R (Darzi et al., 2016). These analyses resulted in 56 new values for each participant regarding each GBM-pathway. The GBMs were then correlated with factors indicative of the fecal microbial composition and abundance, using Spearman’s correlation analyses. Multiple comparisons were adjusted for using the False Discovery Rate (FDR), the Benjamini-Hochberg Procedure.

Associations between selected KEGG Modules connected to the metabolism of macronutrients and stress and depression symptoms were performed with Analysis of Compositions of Microbiomes with Bias Correction (ANCOM-BC2) (Lin and Das Peddada, 2020a), adjusting for sugar intake frequency. Modules not known to occur in bacteria or archaea were excluded.

#### 2.2.4 Statistical software

Statistical analyses were performed using R (v.3.5.2) or IBM SPSS Statistics (Statistical Package for Social Sciences, SPSS, USA) version 27. Logistic regressions were performed with a 95% confidence interval.

## 3 Results

### 3.1 Sample characteristics by mental health

Background characteristics of the participating women are presented according to low/high perceived stress and low/high depressive symptoms, respectively in **Table 1**. There were no significant differences in age, educational level, Body Mass Index (BMI), smoking habits, physical exercise, hormonal contraceptive use, and average plasma hormonal levels or use of antidepressants between the low/high depressive symptoms or between low/high perceived stress. However, symptoms of BV were more prevalent in women with high levels of stress and depressive symptoms (p=0.029 and 0.007), while a varied bowel/colonic transit time (varied between fast and slow) was more prevalent in women with high stress levels (p=0.002). Also, participants with high stress levels reported having intercourse more days during the study period compared with those with low stress levels (p=0.021). No difference in intercourse frequency was found between high/low levels of depressive symptoms. Daily sugar intake, defined as consumption of any foods in 13 specified food groups several times a day, was also more common in women with high depressive symptoms and high stress (p=0.008 and 0.031), see **Table 1**.

**Table 1:** Background characteristics of the participating women grouped by normal vs high level of depressive symptom scores (on the Major Depression Inventory; MDI) and normal vs high level of perceived stress scores (on the Perceives Stress Scale; PSS) respectively.

	*Symptoms of stress*			*Symptoms of depression*		
*Background and health variables*	Low stress PSS < 17 (n=118)	High stress PSS ≥ 17 (n=42)	p-value	No depressive symptoms MDI ≤18 (n=143)	Depressive symptoms MDI >18 (n=17)	p-value
*Age in years; median (range)*	23 (19-40)	23 (18-40)	0.654 ^1^	23 (18; 40)	23 (21; 40)	0.592 ^1^
*Higher education (university level), n(%) vs Lower*	107 (90.7)	38 (90.5)	1.000 ^2^	129 (90.2)	16 (94.1)	1.000 ^2^
*BMI (kg/m^2^; median (range))*	21.8 (18-37)	22.6 (18-29)	0.137 ^1^	22 (18; 37)	18 (23; 28)	0.060 ^1^
*Symptoms of bacterial vaginosis (BV) ^4^, n (%) * *One symptom*	7 (5.9)	7 (16.7)	0.029* ^2^	11 (7.7)	3 (17.6)	0.007*^2^
*Two symptoms*	1 (0.8)	2 (4.8)		1 (0.7)	2 (11.8)	
*Use of antidepressants (SSRIs)*	3 (2.5)	1 (2.4)	0.954	4 (2.8)	0 (0)	0.485
*Sex hormones and vaginal microbiota dynamics (VCD)*							
*Hormonal contraceptives * *No * *Combined Oral contraceptives* *Hormonal Intrauterine Device*	42 (35.6) 34 (28.8) 42 (35.6)	12 (28.6) 18 (42.9) 12 (28.6)	0.248	48 (33.6) 44 (30.8) 51 (35.7)	6 (35.3) 8 (47.1) 3 (17.6)	^ ^ ^ ^ 0.257
*Average Estradiol, median^5^*	.15 (0-1)	.28 (0-1)	0.161^1^	.35 (0.1-0.8)	.28 (0.2-0.6)	0.445^1^
*Average Progesterone, median*	12.1 (0.8-28.8)	11.9 (1.3-23.1)	0.718^1^	12.0 (0.8-28.8)	13.0 (1.4-28.6)	0.314^1^
*Average Progesterone to Estradiol ratio, median^5^*	30.8 (1.9-83.1)	30.6 (2.3-79.2)	0.529^1^	30.3 (2.0-83.1)	45.7 (7.4-79.2)	0.112^1^
*Vaginal community dynamics (VCD) * *Constant eubiotic* *Menses dysbiotic* *Unstable* *Constant dysbiotic*	37 (31.9) 10 (8.6) 23 (19.8) 46 (39.7)	16 (39.0) 5 (12.2) 11 (26.8) 9 (22.0)	0.235^3^	46 (32.9) 13 (9.3) 29 (20.7) 52 (37.1)	7 (41.2) 2 (11.8) 5 (29.4) 3 (17.6)	0.462^3^
*Health behaviors*							
*Alcohol, n(%)* *≥ 8 drinks per week*	36 (30.5)	8 (19)	0.153 ^3^	42 (29.4)	2 (11.8)	0.157 ^2^
*Smoking; yes, n(%)*	35 (29.7)	11 (26.2)	0.670^3^	43 (30.1)	3 (17.6)	0.399 ^2^
*Exercise, n (%) * *Hard exercises several times/week*	14 (11.9)	2 (4.8)	0.235^2^	14 (9.8)	2 (11.8)	0.182^2^
*Medium exercise ≥ 4 hours/week* *No exercise*	103 (87.3) 1 (0.8)	39 (92.9) 1 (2.4)		128 (89.5) 1 (0.7)	14 (82.4) 1 (5.9)	
*Days with intercourse*	4 (0-20)	2 (0-16)	0.021* ^6^	3.5 (0-20)	3 (0-12)	0.403^6^
*Dietary habits*							
*Fibers, n (%) * *Daily* *Less often*	104 (90.4) 11 (9.6)	36 (87.8) 5 (12.2)	0.765^3^	127 (91.4) 12 (8.6)	13 (76.5) 4 (23.5)	0.077 ^3^
*Sugar intake, n (%) * *Daily* *Weekly* *Seldom/never*	9 (7.6) 90 (76.3) 19 (16.1)	11 (26.2) 26 (61.9) 5 (11.9)	0.008*^3^	25 (10.5) 108 (75.5) 20 (14.0)	5 (29.4) 8 (47.1) 4 (23.5)	0.031*^3^
*Bowel/colonic transit time^7,8^, n (%)*							
*Normal colonic transit (BSS 3-4)*	52 (44.1)	10 (23.8)	Reference	55 (38.5)	7 (41.2)	Reference
*Slow colonic transit (BSS 1-2)*	19 (16.1)	6 (14.3)	0.394	23 (16.1)	2 (11.8)	0.650
*Fast colonic transit (BSS 5-7)*	33 (28.1)	13 (31.0)	0.132	43 (30.1)	3 (17.6)	0.403
*Various transit both fast and slow*	14 (11.9)	13 (31.0)	0.002*	22 (15.4)	5 (29.4)	0.363

^1^Mann-whitney U test.

^2^Fisher’s Exact test.

^3^Chi square test.

^4^BV symptoms were reports of “Unpleasant vaginal odor” and “vaginal discharge”.

^5^For the estradiol analyses, women using oral contraceptives and women suing IUS with no menstrual cycle were excluded.

^6^Student’s t-test.

^7^Bristol Stool Form Scale (BSFS).

^8^Analyzed with binary logistic regression with normal colonic transit as the Reference.

*P<0.05

### 3.2 Gut microbiota composition in association with mental health

**Figure 1:**
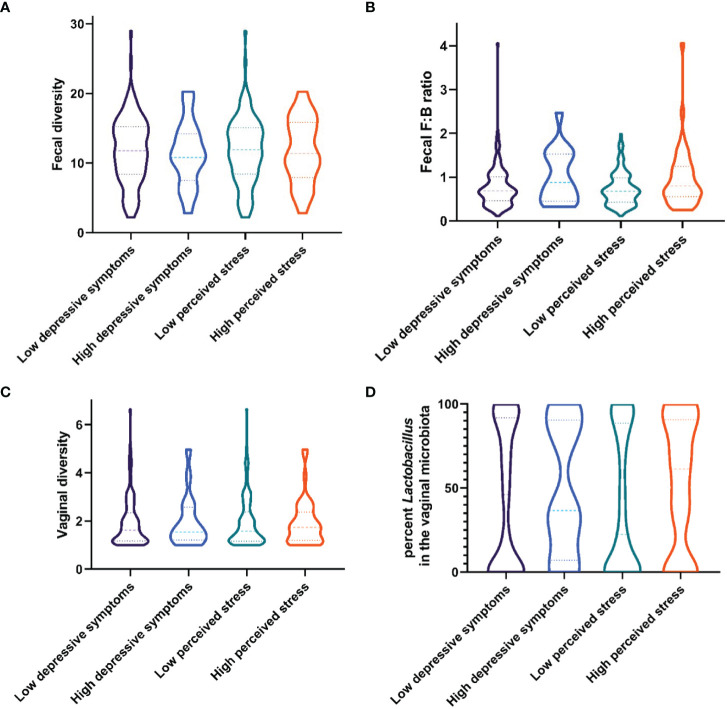
Violin plots showing median levels and interquartile ranges of the microbial composition in women with low or high levels of depressive symptoms and symptoms of perceived stress, respectively. **(A)** Fecal diversity **(B)** Phylum Firmicutes to Bacteroidetes (F/B) ratio **(C)** Vaginal diversity **(D)** Percent of microbiota belonging to the Lactobacillus species.

Median levels and interquartile range of fecal diversity and fecal F/B ratio between groups (low/high depressive symptoms and low/high perceived stress) are presented in **Figures 1A, B**. The odds ratios for the following variables: fecal diversity, fecal richness, and F/B ratio in association with depressive symptoms or high levels of stress are presented in **Tables 2a** and **b**, respectively. None of the microbial composite measures reached significance in the regression models. Daily sugar intake and various bowel/colon transit times were independently associated with high levels of stress, but not with depressive symptoms (**Tables 2a****,** **b**).

**Table 2A:** Unadjusted and adjusted odds ratios representing association of the gut microbiota and symptoms of perceived stress using a cut-off of over 16 points on the Perceived stress scale (PSS).

*Variables*	*Unadjusted*	*Adjusted*
	OR (95% CI)	OR (95% CI)
***Fecal diversity***	1.00 (0.92-1.07)	1.00 (0.92-1.08)
*Sugar intake * *Daily* *Weekly* *Seldom/Never*		**5.54 (1.38-22.33)** 1.02 (0.33-3.15) Reference
*Bowel/colon transit time * *Normal* *Slow* *Fast* *Various slow/fast*		Reference 1.74 (0.53-5.75) 1.79 (0.67-4.80) **8.48 (2.69-26.78)**
***Fecal richness***	1.00 (0.99-1.00)	0.99 (0.99-1.00)
*Sugar intake * *Daily* *Weekly* *Seldom/Never*		**6.33 (1.51-26.57)** 1.06 (0.33-3.41) Reference
*Bowel/colon transit time * *Normal* *Slow* *Fast* *Various slow/fast*		** ** Reference 1.85 (0.55-6.19) 1.75 (0.64-4.75) **6.41 (2.18-18.84)**
***Fecal F.B ratio***	2.03 (0.74-5.58)	2.45 (0.80-7.53)
*Sugar intake * *Daily* *Weekly* *Seldom/Never*		**5.42 (1.33-22.10)** 0.99 (0.31-3.10) Reference
*Bowel/colon transit time * *Normal* *Slow* *Fast* *Various slow/fast*		Reference 1.74 (0.52-5.82) 1.91 (0.70-5.12) **6.62 (2.24-19.55)**

Significant results in bold.

**Table 2B:** Unadjusted and adjusted odds ratios representing association of the gut microbiota and depressive symptoms using a cut-off of over 18 points on the Major Depression Inventory MDI.

Variables	Unadjusted	Adjusted
	OR (95% CI)	OR (95% CI)
***Fecal diversity***	0.98 (0.88-1.09)	0.96 (0.89-1.11)
Sugar intake *Daily* *Weekly* *Seldom/Never*		2.34 (0.49-11.11) 0.37 (0.10-1.41) Reference
Bowel/colon transit time *Normal* *Slow* *Fast* *Various slow/fast*		Reference 0.69 (0.13-3.78) 0.45 (0.10-2.03) 2.39 (0.64-8.95)
***Fecal richness***	1.00 (0.99-1.00)	1.00 (0.99-1.00)
Sugar intake *Daily* *Weekly* *Seldom/Never*		2.61 (0.53-12.91) 0.39 (0.10-1.50) Reference
Bowel/colon transit time *Normal* *Slow* *Fast* *Various slow/fast*		Reference 0.70 (0.13-3.82) 0.51 (0.11-2.23) 2.51 (0.66-9.56)
***Fecal F/B ratio***	2.03 (0.58-7.14)	2.05 (0.52-8.18)
Sugar intake *Daily* *Weekly* *Seldom/Never*		2.18 (0.45-10.53) 0.36 (0.10-1.38) Reference
Bowel/colon transit time *Normal* *Slow* *Fast* *Various slow/fast*		Reference 0.85 (0.15-4.76) 0.49 (0.11-2.23) 2.81 (0.71-11.08)

Significant results in bold.

### 3.3 Vaginal microbiota is not associated with symptoms of stress and depression, but self-reported vaginal symptoms show associations with mental health

The vaginal microbiome diversity and the relative abundance of the most common vaginal species, *Lactobacillus* spp (excluding *L.iners*) between groups (low/high depressive symptoms and low/high perceived stress) are presented in **Figures 1 C, D**. Neither diversity nor non-*Iners Lactobacillus* spp. abundance were significantly correlated with either stress or depressive symptoms (**Supplementary Tables 1a, b**). However, symptoms of BV were associated with higher odds of both stress and depressive symptoms. To further investigate potential differences in the relative abundance of specific bacteria related to BV in stress and/or depression, tests were also performed looking at five groups of vaginal bacterial dominance using a 60% cut-off; *L. crispatus*, *L. iners*, other *Lactobacillus* spp., *G. vaginalis*, *Prevotella* spp. and Mixed (no dominant group). No differences were found for the low/high psychological symptom groups.

**Figure 2:**
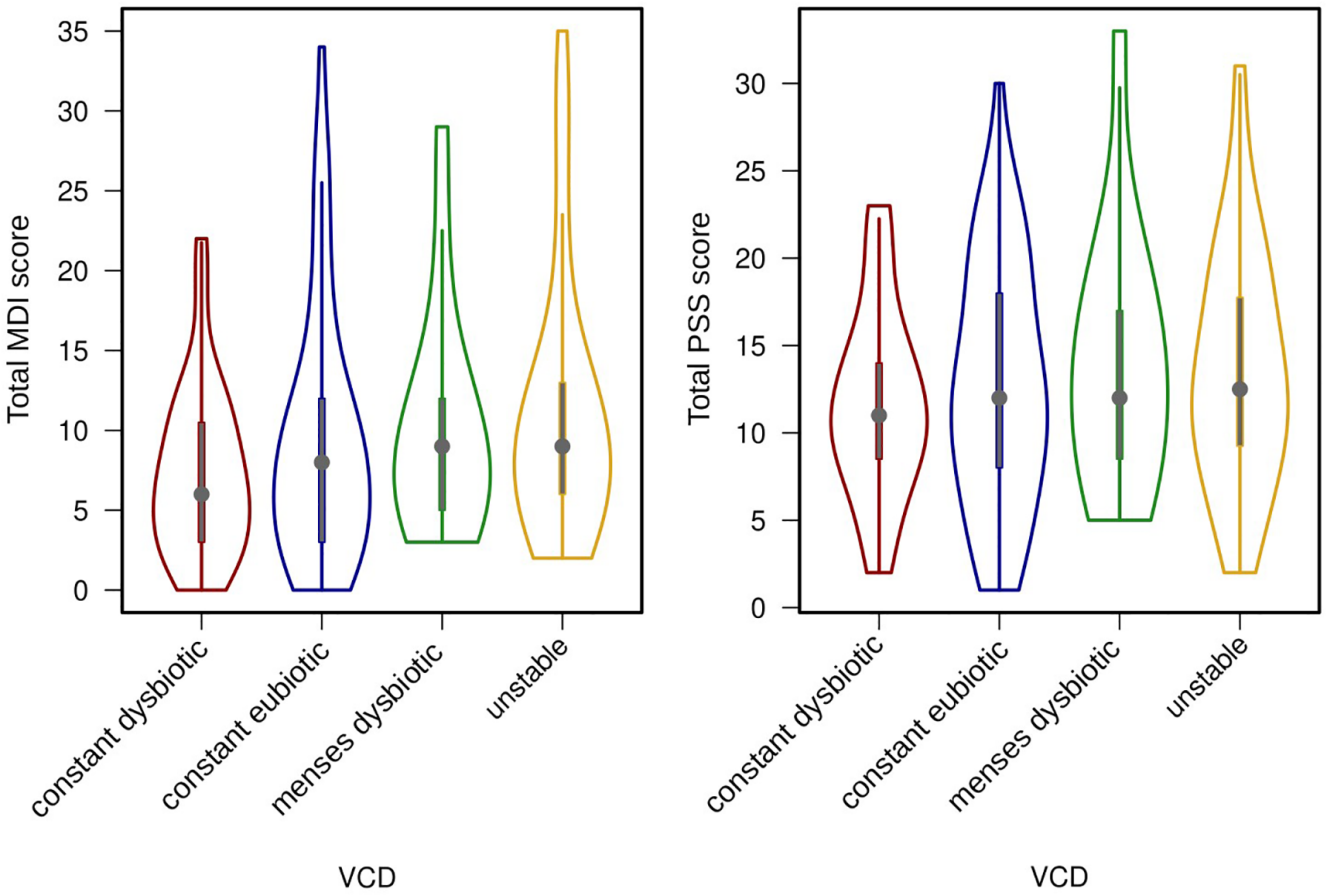
Violin plots showing median levels and interquartile ranges of depressive symptoms (MDI score) and stress (PSS score), divided by their vaginal community dynamics (VCD). Differences between groups are not significant.

To move beyond a single sample, the participant’s vaginal community dynamics (VCD) were classified as “constant eubiotic”, “constant dysbiotic”, “menses dysbiotic” and “unstable” (Hugerth et al., 2023). These dyanamics were not correlated to neither reported levels of stress nor depression, whether we considered the variables categorically (chi-square, p > 0.1) or continuously (ANOVA, p > 0.4), see **Figure 2**. There was however a weak negative correlation between days of reported sexual intercourse and mood (Pearson’s product moment; PSS: r = -0.21, p = 0.01; MDI: r = -0.19, p = 0.02).

### 3.4 Abundance of specific gut bacterial taxa in association with symptoms of stress/depression

**Figure 3:**
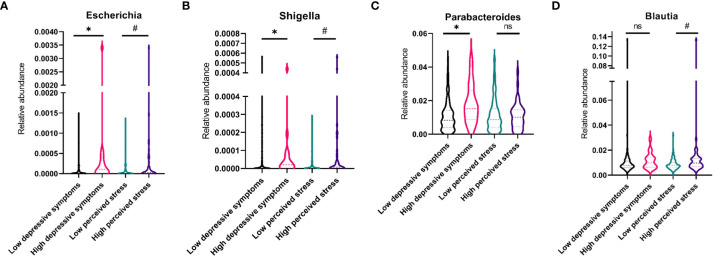
Violin plots showing median levels and interquartile ranges of four fecal bacterial genera in women with low or high levels of depressive symptoms and symptoms of perceived stress, respectively. **(A)** Abundance of Escherichia **(B)** Abundance of Shigella **(C)** Abundance of Parabacteroides **(D)** Abundance of Blautia. * p<.05; # p<.10; ns, nonsignificant.

A higher median abundance of the genera *Escherichia, Shigella*, and *Parabacteroides* was found in women with high levels of depressive symptoms compared with those with fewer symptoms p<0.05, see **Figures 3A–C**. There was a statistical tendency (p<0.10) for higher median abundances of *Shigella, Escherichia*, and *Blautia* in women with a high perceived stress level (**Figures 3A, B, D**). No associations with psychological symptoms were found for *Clostridium*, *Lachnospiraceae*, *Parasutterella*, *Phascolarctobacterium*, *Ruminococcus*, *Bacteroides*, *Oscillibacter*, *Prevotella*, *Roseburia*, or *Actinobacteria*.

### 3.5 Gut metabolic pathways in association with symptoms of stress/depression

Eleven pathways of macronutrient metabolism including amino acids, fatty acids and the citrate cycle were found to be altered in women with high stress levels, and nine pathways were altered in women with high levels of depressive symptoms. The pathways upregulated in stressed individuals were downregulated in those who experienced depressive symptoms and vice-versa (**Supplementary Table 2**). After multiple testing corrections, one module remained significant; the Leucine biosynthesis pathway (2-oxoisovalerate => 2-oxoisocaproate), which was decreased in women with high perceived stress and increased in women reporting high levels of depressive symptoms.

### 3.6 The gut microbiota composition and abundance of bacterial taxa display different functionality relevant for the microbiota-gut-brain axis

**Figure 4:**
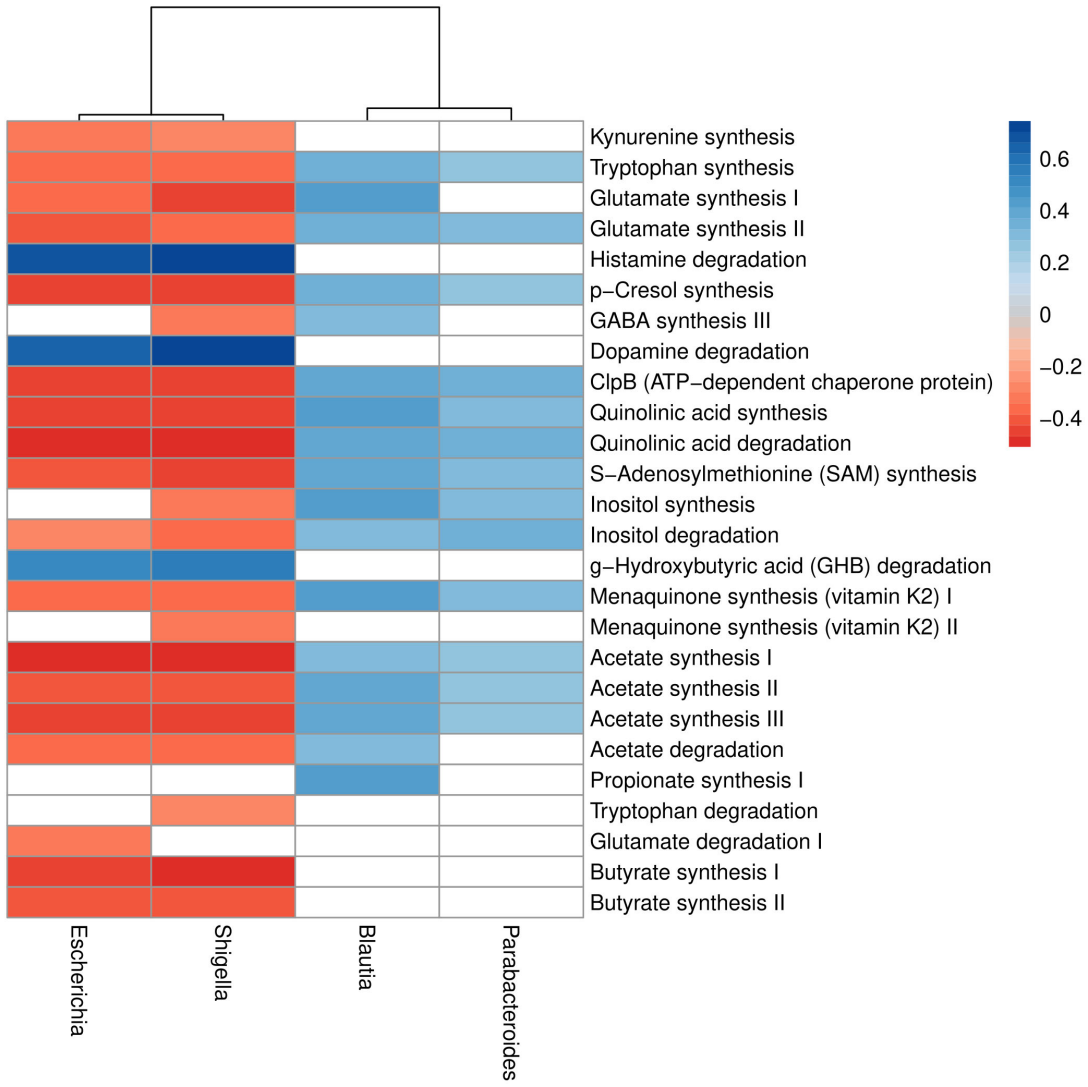
Different gut-brain modules associate to different bacterial phyla and genera. The r-value of Spearman’s rank correlation between gut-brain modules and bacterial genera is depicted as a color scale, with positive correlations in blue and negative correlations in red. Non-significant correlations are left white. Columns are clustered by average-linkage on Spearman’s coefficient, while rows are displayed alphabetically.

Of the 56 gut-brain modules investigated using the KEGG annotation, 42 were detected in the current data and tested for associations with the specific taxa associated or trending with symptoms of stress and depression (p<0.10). These analyses showed that Proteobacteria Escherichia and Shigella, which were positively related to psychological symptoms, were associated with decreased synthesis of kynurenine, tryptophan, glutamate, vitamin K2, acetate and butyrate (see **Figure 4**). Those genera were also associated with lower degradation of dopamine, histamine, and G-hydroxybutyric acid (GHB). Shigella was further associated with decreased Gamma-aminobutyric acid (GABA) synthesis and tryptophan degradation. The Bacteroidetes Parabacteroides (also increased in those with depressive symptoms) and Firmicutes Blautia (increased in stressed individuals; p<0.10) were associated with putative beneficial functions, such as increased synthesis of tryptophan, GABA, vitamin K2, and propionate (**Figure 4**).

## 4 Discussion

### 4.1 Bacterial abundance in the gut and psychological symptoms in healthy women

This study investigated symptoms of stress and depression in association with gut and vaginal microbiota composition and functionality in a cohort of healthy women aged 18-40 years. Our results support previous findings from psychiatric populations. However, fewer genera and no composite microbial variables were found to be associated with depressive symptoms and perceived stress levels. Our findings in this healthy cohort of women could be evaluated as early signs of future psychiatric conditions. In this study, women with high levels of depressive symptoms had a significantly higher relative abundance of the genera *Escherichia*, *Shigella* (Proteobacteria), and *Parabacteroides* (Bacteroidetes), similar to reports in women diagnosed with depression (Chen et al., 2021)*. Escherichia* and *Shigella* have been suggested to induce inflammation by secreting exotoxins, affecting the brain, behavior, and mood (Jiang et al., 2018). Thus, their overgrowth might induce a negative mood. *Escherichia coli* has also been shown to induce negative mood-like behaviors in rats (Jaglin et al., 2018). However, in a study of depressed men and women where the majority were treated with antidepressants, a lower abundance of *Escherichia/Shigella* was found (Jiang et al., 2015). A diet with low fiber and high sugar intake increases the growth of *Escherichia coli* (Jian et al., 2021), which could further increase the negative mood in a vicious cycle (Kim et al., 2020). The connection between a decreased metabolism of sucrose and carbohydrates in depressed individuals has been highlighted recently (Zheng et al., 2016; Chung et al., 2019), but the question remains whether this is a driver or a consequence of the disease. When analyzing the metabolic pathways of the gut microbiome in this study, no associations with the metabolism of sucrose or carbohydrates were found, only with amino acids, fatty acids, and the citrate cycle in the unadjusted analyses. In women with high levels of perceived stress, a high relative abundance of *Blautia* (Firmicutes) was identified, in line with earlier findings (Chung et al., 2019).

In contrast to many previous studies, fecal diversity, richness and the F/B ratio did not correlate with psychological symptoms in this healthy cohort of women. Importantly, our data was transformed using a centered-log ratio procedure to decrease the rate of false positive findings (Gloor et al., 2017; Lin and Das Peddada, 2020b).

### 4.2 Abundance of specific gut taxa and associated functionality

We investigated the genetic functional repertoire of the different taxa and found associations with 26 GBMs (Valles-Colomer et al., 2019). Of particular interest, alterations in the glutamatergic/GABAeric pathways, dopaminergic, and Kynurenine pathways are linked to bacteria associated with stress and depressive symptoms in this study. The increase in *Escherichia* and *Shigella* was related to a decrease in the microbiota-gut-brain function of tryptophan synthesis and a similar decrease in GABA synthesis associated with *Shigella* only. *Shigella* and *Escherichia* are often grouped together, as 16S rRNA gene sequencing cannot distinguish these genera. Interestingly, *Escherichia coli* has recently been shown to activate pathways involved in the degradation of neurotransmitters gamma-aminobutyric acid (GABA) and serotonin (Joffré et al., 2021). *Escherichia* and *Shigella* were also associated with a decrease in kynurenine synthesis as well as quinolinic synthesis and degradation. It should be noted that the genera *Parabacteroides* (increased in association with depressive symptoms) and *Blautia* (tendency to be higher in stressed individuals) seem to promote pathways protective of affective disorders.

Another finding was a decrease in the leucine biosynthesis pathway (2-oxoisovalerate => 2-oxoisocaproate) in women with higher levels of perceived stress. Leucine is an essential branched-chain amino acid (BCAA). While not generally considered neuroactive, BCAA in general, and leucine, in particular, have mood regulatory effects in mice, including protection against stress and depression (Walker et al., 2019; Nasrallah et al., 2019). In a cross-sectional study, a similar observation was made in humans (Koochakpoor et al., 2021), and blood levels of BCAAs have been linked to brain levels of tryptophan (Blomstrand, 2006).

In addition, an increase in the genera *Escherichia* and *Shigella* was associated with a decrease in the functional pathway of glutamate synthesis. Excessive activation of glutamate receptors could be involved in several psychiatric conditions (reviewed in (Haroon and Miller, 2017)) and glutamate in the gut can influence brain function (Zhou and Danbolt, 2014; Onaolapo and Onaolapo, 2021). An association between higher abundance of *Escherichia* and *Shigella* was associated with lower function in the acetate synthesis, another actor in the glutamate/GABA–glutamine cycle (Rowlands et al., 2017), while *Blautia* and *Parabacteroides* support acetate synthesis. A decrease in acetate synthesis was previously seen in depressed women compared with controls (Skonieczna-żydecka et al., 2018a).

### 4.3 Gut and vaginal symptoms in relation to stress/depressive symptoms

Women with high levels of perceived stress more often reported a transit-time-pattern comprising both fast and slow transit, similar to the IBS mixed phenotype (Kim and Kim, 2018). Activation of the gut-brain-axis by stress seems to trigger IBS symptoms (Raskov et al., 2016; Margolis et al., 2021).

Our results did not reveal any link between vaginal microbial diversity, the proportion of *Lactobacillus* spp. nor vaginal community dynamics during their menstrual cycle with reported mental health scores. However, women with high perceived stress more often had vaginal symptoms of BV, supporting a link between stress and less optimal vaginal health (Culhane et al., 2006; Nansel et al., 2006) although without documenting a higher prevalence of vaginal dysbiosis.

### 4.4 Hormonal levels and psychological symptoms

In the present study, plasma levels of estradiol and progesterone did not differ according to levels of depressive symptoms and perceived stress. However, many women were using hormonal contraceptives, which limits the possibility of drawing valid conclusions. Notably, no associations between contraceptive use and psychological symptoms were found, in contrast with previous findings of hormonal contraceptive use being linked to mood disorders (Skovlund et al., 2016; Lundin et al., 2022). However, there was a slight difference in sexual behavior, with a higher frequency of sexual intercourse in the group with low levels of reported perceived stress.

In summary, our results indicated that the gut microbiota may impact significant neuroactive pathways such as the Kynurenine pathway, and GABAergic, glutamatergic, and dopaminergic neurotransmission, even in healthy individuals. This implies, that targeting the gut microbiome could be a promising approach for future psychiatric interventions. This study adds to the literature by applying research in the field of gut-microbiota-brain-axis in a sample of healthy women and by relating microbial composition to functional pathways. It could be hypothesized that stress could alter the gut microbiota as well as eating behaviors, and thereby influencing negative mood pathways. The results of this study in healthy women suggest that an increased abundance of *Escherichia* and *Shigella* may precede mood disturbances. However, more research is needed before microbial testing would be feasible to suggest for clinical routine screening. Promoting a diet rich in fiber and limiting sugar intake to counteract Escherichia coli overgrowth may be relevant in general clinical practice but intervention studies are needed to establish causality and efficacy before implementing such recommendations. In this study, the association between reported unpleasant vaginal symptoms and high levels of perceived stress was not correlated to acuta vaginal dysbiosis and thus warrants further research.

### 4.5 Strengths and limitations

A major strength of this study is the homogenous and well-defined cohort of women. The healthy cohort did not warrant clinical diagnoses and the mental health questionnaires were all validated and are used both clinically and in research settings. However, the direct applicability to clinical populations is limited. The relatively small sample size highlights the importance of replicating the findings in larger cohorts of healthy women. This study adds the novelty of investigating vaginal microbiota in relation to mental health, albeit with negative findings. Furthermore, the cross-sectional design hinders investigating the causal mechanisms of mood, behaviors, and gut microbiota composition.

## Data availability statement

The datasets presented in this study can be found in online repositories. The names of the repository/repositories and accession number(s) can be found below: https://www.ebi.ac.uk/ena, PRJEB37731 samples ERS4421369–ERS4422941.

## Ethics statement

The studies involving humans were approved by The Regional Ethics Committee (H-17017580) The Data Protection Agency (RH-2017-280, I-Suite 05825) in the Capital Region of Denmark. The studies were conducted in accordance with the local legislation and institutional requirements. The participants provided their written informed consent to participate in this study.

## Author contributions

ZB: Data curation, Writing – original draft, Investigation, Project administration. LH: Formal analysis, Methodology, Software, Visualization, Writing – review & editing. MK: Data curation, Investigation, Methodology, Project administration, Writing – review & editing. SP-N: Formal analysis, Methodology, Writing – review & editing. GE: Formal analysis, Writing – review & editing. FB: Writing – review & editing, Data curation, Supervision, Software. SS: Data curation, Writing – review & editing. IT: Data curation, Writing – review & editing, Methodology. LE: Writing – review & editing, Conceptualization, Funding acquisition, Resources. IS-K: Writing – review & editing, Conceptualization. EF: Data curation, Formal analysis, Visualization, Writing – original draft. HN: Conceptualization, Funding acquisition, Resources, Supervision, Writing – review & editing.
