# Supplementary material for: Investigations of microbiota composition and neuroactive pathways in association with symptoms of stress and depression in a cohort of healthy women
Source: Front Cell Infect Microbiol. 2024 Jul 2;14:1324794. doi: 10.3389/fcimb.2024.1324794 (PMC11249552; doi:10.3389/fcimb.2024.1324794)
Supplement: Supplementary file 1 [file DataSheet_1.docx]

**Supplementary Table 1a**. Unadjusted and adjusted odds ratios representing association of the vaginal microbiota via Vaginal diversity and Percent Lactobacillus spp, respectively and symptoms of perceived stress using a cut-off of over 16 points on the Perceived stress scale (PSS). Significant results in bold.

|  | Variables | OR (95% CI) | | |
| --- | --- | --- | --- | --- |
|  |  | Unadjusted | | Adjusted |
|  | Vaginal diversity | 0.98 (0.91-1.04) | | 0.98 (0.91-1.05) |
|  | Symptoms of bacterial vaginosis |  | | **2.96 (1.22-7.02)** |
|  | Percent Lactobacillus spp. | 1.00 (0.99-1.02) | 1.00 (0.99-1.02) | |
|  | Symptoms of bacterial vaginosis |  | **2.97 (1.25-7.08)** | |

**Supplementary Table 1b**. Unadjusted and adjusted odds ratios representing association of the vaginal microbiota via Vaginal diversity and Percent Lactobacillus spp, respectively and depressive symptoms using a cut-off of over 18 points on the Major Depression Inventory (MDI). Significant results in bold.

|  | Variables | OR (95% CI) | | |
| --- | --- | --- | --- | --- |
|  |  | Unadjusted | | Adjusted |
|  | Vaginal diversity | 1.05 (0.96-1.14) | | 1.05 (0.99-1.16) |
|  | Symptoms of bacterial vaginosis |  | | **4.03 (1.56-10.46)** |
|  | Percent Lactobacillus spp. | 1.00 (0.97-1.02) | 0.99 (0.97-1.02) | |
|  | Symptoms of bacterial vaginosis |  | **3.91 (1.53-10.01)** | |
