# Supplementary material for: Investigations of microbiota composition and neuroactive pathways in association with symptoms of stress and depression in a cohort of healthy women
Source: Front Cell Infect Microbiol. 2024 Jul 2;14:1324794. doi: 10.3389/fcimb.2024.1324794 (PMC11249552; doi:10.3389/fcimb.2024.1324794)
Supplement: Supplementary file 2 [file DataSheet_2.docx]

**Supplementary Table 2.** Linear models correlating gut-brain modules to a combination of depression (MDI score), and stress (PSS score), adjusting for sugar and fiber intake. All modules with a significant association before multiple testing correction are depicted, but the only module that is still significant after FDR is highlighted in bold. Each module is described as their unique KEGG identifier but also as a functional description. Raw p-values and adjusted p-values (q-values) are shown for MDI and PSS for each module, as well as the full linear model. For example, module M00432 can be described as: M00432 = 0,00564 - 0,00043*PSS + 0,00036*MDI -0,00009*sugar+ 0,00004*fiber.

| **Module ~ MDI + PSS + sugar + fibre** | |  |  |  |  | **Model** | | | | |
| --- | --- | --- | --- | --- | --- | --- | --- | --- | --- | --- |
| **Module** | **Module description** | **PSS pval** | **PSS qval** | **MDI pval** | **MDI qval** | **Intercept** | **PSS** | **MDI** | **sugar** | **fiber** |
| M00004 | Pentose phosphate pathway (Pentose phosphate cycle) | 0,016 | 0,181 | 0,025 | 0,358 | 0,008024 | 0,00047 | -0,00052 | 0,00003 | -0,00007 |
| M00006 | Pentose phosphate pathway, oxidative phase, glucose 6P => ribulose 5P | 0,028 | 0,223 | 0,028 | 0,358 | 0,002355 | 0,00030 | -0,00035 | -0,00007 | -0,00004 |
| M00008 | Entner-Doudoroff pathway, glucose-6P => glyceraldehyde-3P + pyruvate | 0,028 | 0,223 | 0,032 | 0,358 | 0,002718 | 0,00027 | -0,00031 | -0,00009 | 0,00003 |
| M00011 | Citrate cycle, second carbon oxidation, 2-oxoglutarate => oxaloacetate | 0,061 | 0,377 | 0,010 | 0,358 | 0,011723 | -0,00059 | 0,00090 | 0,00017 | -0,00027 |
| M00016 | Lysine biosynthesis, succinyl-DAP pathway, aspartate => lysine | 0,008 | 0,127 | 0,065 | 0,430 | 0,014642 | -0,00041 | 0,00033 | -0,00011 | -0,00001 |
| M00018 | Threonine biosynthesis, aspartate => homoserine => threonine | 0,008 | 0,127 | 0,050 | 0,368 | 0,009880 | -0,00025 | 0,00021 | 0,00014 | 0,00007 |
| M00027 | GABA (gamma-Aminobutyrate) shunt | 0,003 | 0,085 | 0,048 | 0,368 | 0,001099 | 0,00015 | -0,00014 | -0,00002 | -0,00005 |
| M00036 | Leucine degradation, leucine => acetoacetate + acetyl-CoA | 0,132 | 0,468 | 0,023 | 0,358 | 0,002193 | -0,00009 | 0,00017 | 0,00005 | 0,00007 |
| M00114 | Ascorbate biosynthesis, plants, glucose-6P => ascorbate | 0,126 | 0,468 | 0,043 | 0,368 | 0,007807 | -0,00023 | 0,00038 | -0,00018 | 0,00013 |
| **M00432** | **Leucine biosynthesis, 2-oxoisovalerate => 2-oxoisocaproate** | 0,000 | 0,013 | 0,027 | 0,358 | 0,005640 | -0,00043 | 0,00036 | -0,00009 | 0,00004 |
| M00525 | Lysine biosynthesis, acetyl-DAP pathway, aspartate => lysine | 0,016 | 0,181 | 0,427 | 0,793 | 0,012984 | -0,00025 | 0,00010 | 0,00000 | -0,00007 |
| M00526 | Lysine biosynthesis, DAP dehydrogenase pathway, aspartate => lysine | 0,026 | 0,223 | 0,148 | 0,687 | 0,011693 | -0,00032 | 0,00024 | -0,00007 | -0,00006 |
| M00527 | Lysine biosynthesis, DAP amino-transferase pathway, aspartate => lysine | 0,043 | 0,309 | 0,195 | 0,793 | 0,014609 | -0,00035 | 0,00028 | -0,00017 | -0,00008 |
| M00535 | Isoleucine biosynthesis, pyruvate => 2-oxobutanoate | 0,003 | 0,085 | 0,013 | 0,358 | 0,002266 | -0,00026 | 0,00031 | -0,00006 | -1,28700 |
